# Designing Rigid DNA Origami Templates for Molecular Visualization Using Cryo-EM

**DOI:** 10.1021/acs.nanolett.4c00915

**Published:** 2024-04-11

**Authors:** Ali Khoshouei, Georg Kempf, Volodymyr Mykhailiuk, Johanna Mariko Griessing, Maximilian Nicolas Honemann, Lukas Kater, Simone Cavadini, Hendrik Dietz

**Affiliations:** † Laboratory for Biomolecular Nanotechnology, Department of Biosciences, School of Natural Sciences, Technical University of Munich, Am Coulombwall 4a, 85748 Garching, Germany; ‡ Munich Institute of Biomedical Engineering, Technical University of Munich, Boltzmannstraße 11, 85748 Garching, Germany; § Friedrich Miescher Institute for Biomedical Research, Maulbeerstrasse 66, 4058 Basel, Switzerland

**Keywords:** DNA origami, Design Optimization, Cryo-EM, Scaffolding, Thrombin Binding Aptamer

## Abstract

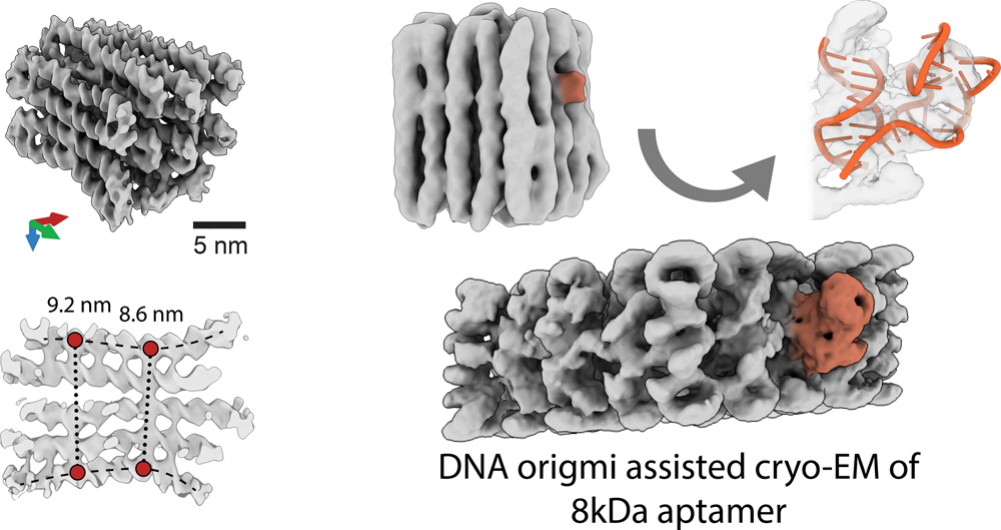

DNA origami, a method for constructing nanostructures from DNA, offers potential for diverse scientific and technological applications due to its ability to integrate various molecular functionalities in a programmable manner. In this study, we examined the impact of internal crossover distribution and the compositional uniformity of staple strands on the structure of multilayer DNA origami using cryogenic electron microscopy (cryo-EM) single-particle analysis. A refined DNA object was utilized as an alignment framework in a host–guest model, where we successfully resolved an 8 kDa thrombin binding aptamer (TBA) linked to the host object. Our results broaden the spectrum of DNA in structural applications.

DNA origami^1^ is a rapidly evolving field in nanotechnology that has shown great promise for creating nanostructures created by folding a long single-stranded DNA “scaffold” into a desired shape using hundreds of short, synthetic DNA strands called staples. The ability to design and build custom nanostructures with DNA origami has opened new opportunities in fields such as biomedicine, nanoelectronics, and materials science.^2−11^ One of the popular uses for DNA origami is to act as a support to place other non-DNA functionalities at predefined spatial locations. The placement accuracy may be affected by compositional and structural heterogeneity, including defects and instability in the DNA origami supports,^7,12,13^ which can result from variations in the purity of the starting materials and the complex nature of the DNA origami fabrication process. To enable further progress in precision placement of molecular functionalities on DNA origami, we evaluated structural aspects of DNA origami objects as a function of design parameters and staple strand purity.^6,14,15^ Specifically, we focused on the structural effects of variations in crossover density, staple length, and staple strand purity, aiming at identifying solutions that yield more accurately defined structures.

Single-particle analysis with cryo-EM (SPA) is a powerful imaging technique that can provide high-resolution structural information on biological macromolecules such as proteins and their complexes under nearly native conditions.^16−19^ SPA has previously been utilized for visualizing the three-dimensional structure of DNA origami at resolutions that allow discerning individual helices and helical details such as major and minor grooves.^20−23^ Here we used SPA to systematically investigate the influence of design modifications and material homogeneity on DNA origami’s folding behavior and structure.

Cryo-EM images of biological samples commonly suffer from a low signal-to-noise ratio, limiting the range of protein sizes that can be studied with SPA.^24^ Small biomolecules with molecular weights below 100 kDa may be difficult to analyze with SPA because the low signal-to-noise ratio in cryo-EM images can obscure important structural features that are needed for accurate alignment and averaging of particle images, which are crucial steps in the SPA process. Poor alignment and averaging of particles can result in a loss of high-resolution information, negatively impacting the final reconstructed map.^25^ A previous analysis estimates the lower-molecular-weight limit for the single-particle cryo-EM study of individual protein molecules to be around 38 kDa.^26^ As a result, determining small biomolecular structures via cryo-EM has been a persistent challenge in the field, and alternative strategies have been investigated to improve the SPA analysis. Scaffolding (i.e., fixing the target of interest to larger support structures providing improved contrast) has been explored with proteins for the structural determination of macromolecular assemblies using cryo-EM. Recent developments in protein design have enabled the creation of geometric protein assemblies, such as cubic cages or clusters.^27,28^ These designed assemblies can serve as “host” scaffolds for attaching smaller “guest” proteins to facilitate cryo-EM imaging.^29,30^ In previous work, two challenges were successfully addressed: linking guest proteins through continuous α-helical linkers between the host scaffold and guest protein and introducing modularity by engineering an adaptor module based on DARPin. The host scaffolds were based on designed assemblies with cubic symmetry, which offer advantages for data processing and overcoming the problem of preferred orientation in cryo-EM. The structures of the host–guest complex could be determined with approximately 3.8 Å resolution using GFP, a 26 kDa guest protein.^30^ DNA origami support scaffolds have also been previously considered for helping to determine the structure of proteins in cryo-EM,^31,32^ offering an alternative platform for nanoscale host structure assembly. Here, we add to these efforts by evaluating the utility of an improved DNA origami object to help solve the structure of nucleic acid-based target molecules.

We generated three distinct variants of a multilayer DNA origami, denoted as V1, V2, and V3 (Supporting Information, Supplementary Figures 1–3), each utilizing a custom scaffold DNA single strand with a length of 2873 bases (Supporting Information, Supplementary Note 1).^33^ The three objects were designed using cadnano software, employing a square lattice helical packing configuration.^6^ While we maintained the overall shape of the DNA origami across the variants, we introduced specific changes to the design parameters, as illustrated schematically in Figure 1A. V1 and V2 differ primarily in the staple strand length distribution (Figure 1B). V1 was designed using staple lengths ranging from 40 to 80 nucleotides, with an average length of 67 nucleotides. In contrast, V2 incorporated staple lengths ranging from 40 to 60 nucleotides, with an average length of 54. V2 also exhibited significantly more scaffold crossovers than V3 (138 vs 86). The three versions were self-assembled as previously described and subjected to an agarose-gel electrophoretic mobility analysis (Figure 1C). V2 exhibited electrophoretic mobility enhancement relative to the other two variants, indicating that V2 had a more compact overall shape. V1 and V2 had similar extents of folding byproducts, whereas V3 featured the fewest byproducts. To perform cryo-EM analysis, we purified all three DNA origami samples from excess staple strands and increased their concentration using PEG precipitation^34^ and molecular weight cutoff filtration (Supporting Information, Supplementary Figure 10). We imaged all three samples in a 300 kV Titan Krios instrument and performed cryo-EM SPA to determine the three-dimensional cryo-EM structure for each design variant. Figures 1D provides representative cryo-EM 2D classes, and Figure 1E gives Fourier-shell correlation (FSC) plots of the final reconstructed 3D volumes. The thus-determined cryo-EM density maps reveal the overall shape and organization of the DNA origami objects and have sufficient details to discern interhelical crossovers and individual helices, including major–minor groove features (Figure 1F). The final resolutions achieved in V1, V2, and V3 are 8.6, 7.5, and 8.3 Å, respectively. Considering that the cryo-EM data sets were collected and processed under equivalent conditions using the same microscope, camera, software, and depth of data, the higher resolution observed in V2 may indicate that it possesses less flexibility than the other variants.

**Figure 1:**
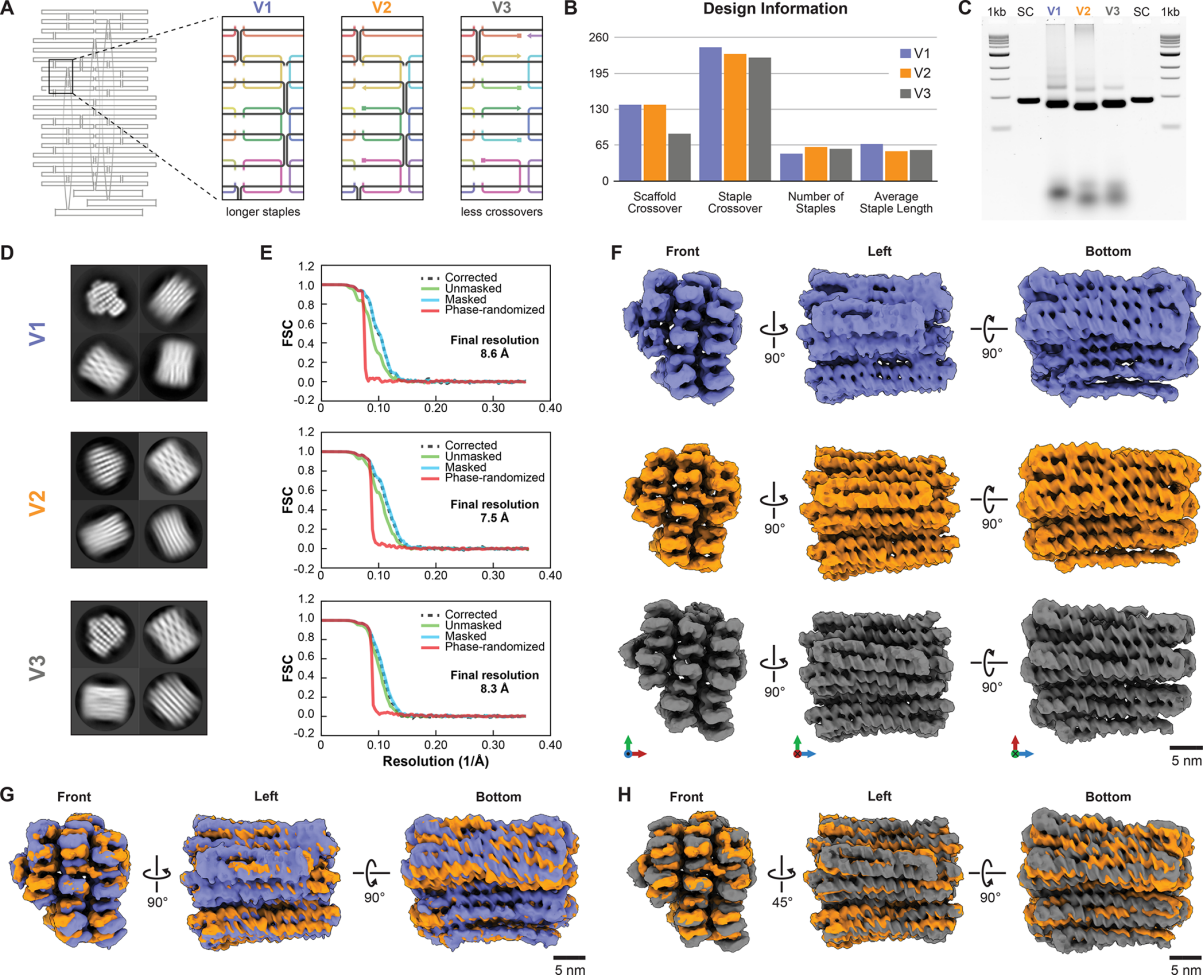
Impact of design parameters on DNA origami compactness. A) Three unique DNA origami variants were created, each featuring differences in the number of scaffold and staple crossovers, the total number of staples, and the average lengths. The magnified area exemplifies these variations, demonstrating the changes consistently applied throughout each variant’s entire structure. B) Detailed design statistics highlighting the variations among the DNA origami designs. C) Gel electrophoresis analysis demonstrating differential migration speeds for the three DNA origami samples. D) Cryo-EM 2D classification results showcasing the structural diversity within each DNA origami variant. E) FSC curves indicate the final resolutions of the 3D reconstructed DNA origami data. The resolutions obtained were 8.6 Å for V1, 7.5 Å for V2, and 8.3 Å for V3. F) 3D reconstructed DNA origami models presented from various viewpoints to visualize different perspectives. G) Comparative analysis of the compactness between V1 and V2 achieved by overlaying the cryo-EM maps on each other. H) A comparative analysis of compactness between V2 and V3 was accomplished by comparing the cryo-EM maps and showcasing different views.

We aligned the cryo-EM maps to compare the compactness of the designed DNA origami structures. The map determined for V2 is more compact than those determined for V1 and V3, as judged by the interhelical lattice spacing (Figure 1G,H, respectively). These observations are consistent with the previous findings from gel electrophoresis, where V2 had the highest electrophoretic mobility. Accordingly, in line with previous findings on coarser scales,^35^ we attribute the enhanced compactness and improved resolution obtained for V2 to the larger number of crossovers used in V2.

We also investigated the effect of staple strand purity on our ability to determine a high-quality cryo-EM structure. DNA origami objects are commonly made from chemically synthesized staple oligonucleotides, which are prone to synthesis errors, such as truncations. These imperfections could contribute to the heterogeneity of the DNA origami structures. We tested for such influences using an HPLC-purified chemically synthesized DNA staple and staple strands purified by desalting. The sample was a multilayer DNA origami object termed V4 (Supporting Information, Supplementary Figure 4) in square lattice packing produced using a custom scaffold strand with a length of 1033 bases (Supporting Information, Supplementary Note 2). The variant V4 was designed by adhering to the staple strand length distribution and crossover scheme identified for V2. To illustrate the differences in staple strand purity, five randomly selected staple strands were analyzed by using polyacrylamide gel electrophoresis (PAGE) (Figure 2A). Desalted or HPLC-purified samples showed distinct band patterns. The desalted stables exhibited additional bands with increased electrophoretic mobility, indicating truncated DNA fragments. Conversely, the HPLC-purified samples show a major band corresponding to the desired DNA length. This indicates the successful removal of the truncated products (Figure 2A).

**Figure 2:**
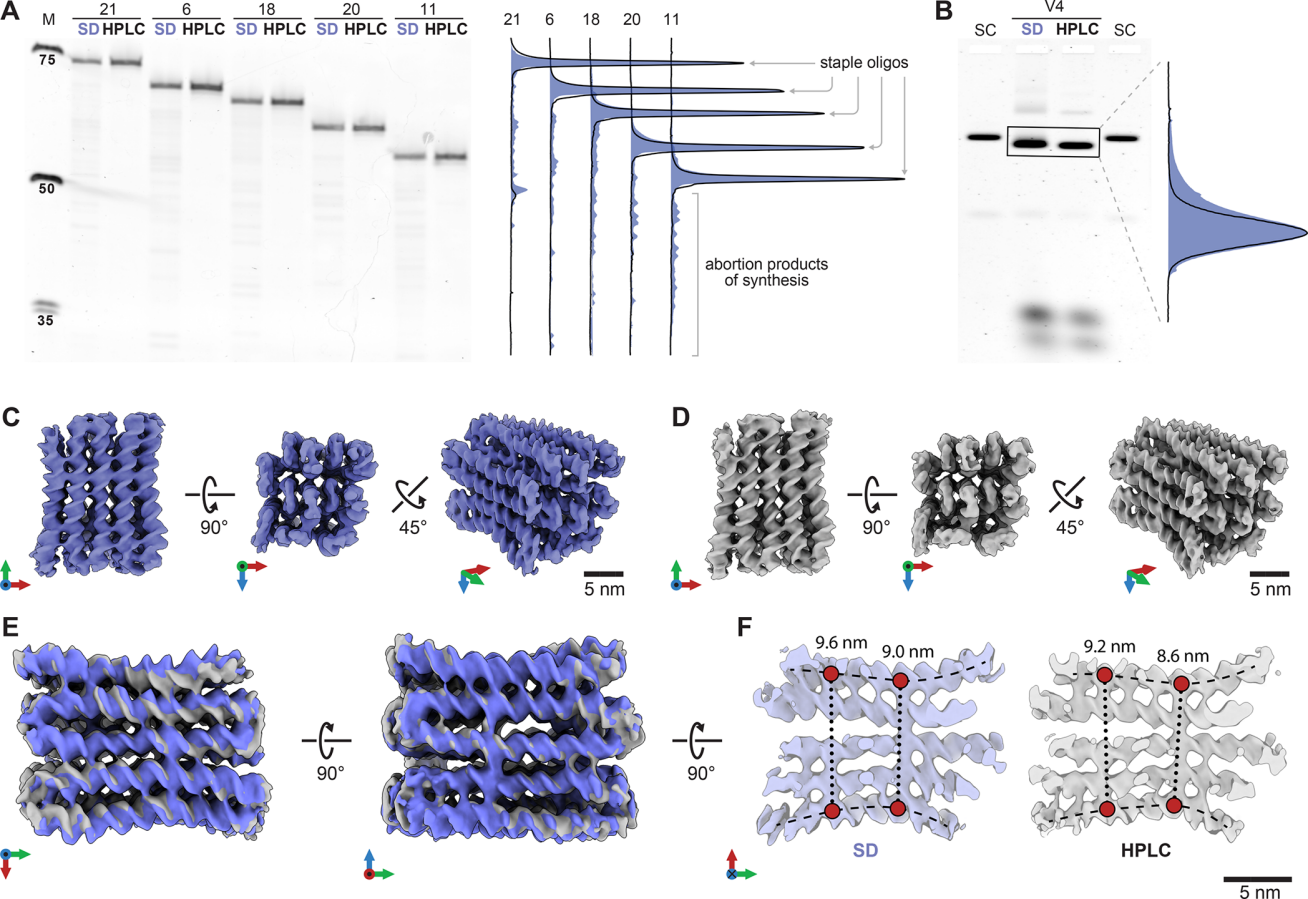
Impact of compositional homogeneity on the compactness of DNA origami. A) Polyacrylamide gel electrophoresis (PAGE) image illustrating the contrast in quality between desalted purified DNA strands (SD) and HPLC-purified oligos. Five representative staple strands of varying length (73, 68, 66, 61, and 56 nucleotides) are presented. B) Gel electrophoresis analysis of DNA origami assemblies employing SD and HPLC-purified staples, revealing discernible distinctions in structural compactness. C) Cryo-EM data set illustrating the folded DNA origami structure utilizing desalted purified staples. The achieved overall structural resolution is 7.8 Å. D) Cryo-EM data set showcasing the folded DNA origami structure using oligos purified through HPLC. The obtained overall structural resolution is 7.2 Å. E) Overlay of cryo-EM reconstructions, accentuating the predominant surface region in various orientations to indicate the compactness level. F) Single-layer DNA origami structure with five parallel helices revealed by longitudinal cross sections. Comparative measurements between the upper and lower helices at two points show a uniform width variation of roughly 4 Å.

To investigate the impact of staple purity on the structure of DNA origami object V4, we performed self-assembly experiments using both desalted and HPLC-purified staples. Gel electrophoresis analysis showed that the HPLC-purified staple strand sample had a clearer, faster-moving, and sharper object band than the sample made with simply desalted staple strands (Figure 2B). Again, we resorted to cryo-EM SPA to determine the structures of the V4 objects assembled from desalted or HPLC-purified staple strands (Figure 2C,D). In the case of the variant assembled from desalted oligonucleotides, the cryo-EM consensus map reached an overall resolution of 7.8 Å, computed from a data set containing 409K particles. The 3D cryo-EM consensus map of the variant constructed with HPLC-purified oligonucleotides achieved an enhanced resolution of 7.2 Å despite a slightly smaller data set of approximately 390K particles.

Multilayer DNA origami objects exhibit internal flexibility, such as lattice breathing or domain motions.^21^ Multibody analysis or focused refinement allows us to consider such flexibility and refine regions of interest with higher resolution.^36^ Since the mostly cubic V4 DNA origami does not exhibit distinct structural domains, we performed the focused refinement with subdomain slices, including peripheral parts and one featuring the central core domain. The refined structures are provided in Supporting Information, Supplementary Figures 11 and 12. The focused refinement led to substantial improvements in resolution, with the central domains of the two V4 object samples reaching comparable overall resolutions of 5 and 5.1 Å in the cases of desalted and HPLC-purified samples, respectively (Supporting Information, Supplementary Figures 11 and 12).

We aligned the two cryo-EM maps to assess potential structural differences resulting from the purification methods. Both data sets underwent identical data processing and acquisition procedures, including applying the same threshold before map fitting. The overlay reveals that the predominant structural features agree closely (Figure 2E). We compared the width of two variants at two points within a single helical layer from longitudinal cross sections. Figure 2F illustrates this width variation measured between the centers of the upper and lower helices. Both points exhibit a difference of approximately 4 Å, suggesting that the HPLC-purified staple origami was on average more compact than its desalted counterpart.

Our gel electrophoresis and cryo-EM SPA data suggest that superior staple strand quality improves assembly and analysis outcomes under otherwise consistent conditions. However, with the focused multibody refinement analysis, the sample made with improved-purity strands lost its advantage. We speculate that the whole-object-level differences may be caused by a small number of additional defects (i.e., a few randomly missing crossovers) that are present in the object made with lower-purity strands. The attainable resolution using multibody refinement may, in turn, be limited by other factors.

We next tested the utility of DNA origami host supports to help resolve small guest targets. We chose a 27-base-long thrombin-binding DNA aptamer^37^ with a molecular weight of approximately 8 kDa. To incorporate the DNA aptamer, we used the DNA origami V4. At selected sites, the scaffold strand was left single-stranded for hybridization with specific handle sequences flanking the DNA aptamer (eight bases at the 3′ end and eight at the 5′ end of the aptamer sequence), and the staples were purified using HPLC (Figure 3A). To test for successful fusion of the aptamer to the DNA origami, the aptamer was labeled terminally with the fluorescent molecule Cy5 (Supporting Information, Supplementary Figure 13) and it retained its activity (Supporting Information, Supplementary Figure 15). The aptamer-origami complexes were purified using size exclusion chromatography, followed by concentrating using molecular-weight-cutoff filtration. Gel electrophoresis analysis confirmed the retention of the aptamer after purification and sample concentration (Supporting Information, Supplementary Figure 14). We used SPA to obtain a DNA origami aptamer complex structure. Figure 3B shows a representative micrograph, 2D classifications, and a consensus map of DNA origami particles at an overall resolution of 7.5 Å. The consensus identified the aptamer as a globular protrusion at the expected site. Multibody refinement was used to improve the resolution of the central domain (4.9 Å), leading to a better-resolved EM density for the DNA aptamer (Figure 3C,D and Supporting Information, Supplementary Figure 16). The map features corresponding to the fused aptamer suggest a twist of the G-quadruplex head toward the stem helix compared to the crystal structure (4I7Y, Figure 3E). As detailed in the methods, a model based on the aptamer crystal structure and double-stranded scaffold DNA could be fitted by restrained molecular dynamics (MDFF). The observed conformational change relative to the reference crystal structure could be due to the absence of the α-thrombin which was cocrystallized in a bound state with the aptamer bound but was absent in our experiments. The 5′ end of the aptamer stem that connects to the scaffold DNA is also expected to form a geometry-restraining U-turn.

**Figure 3:**
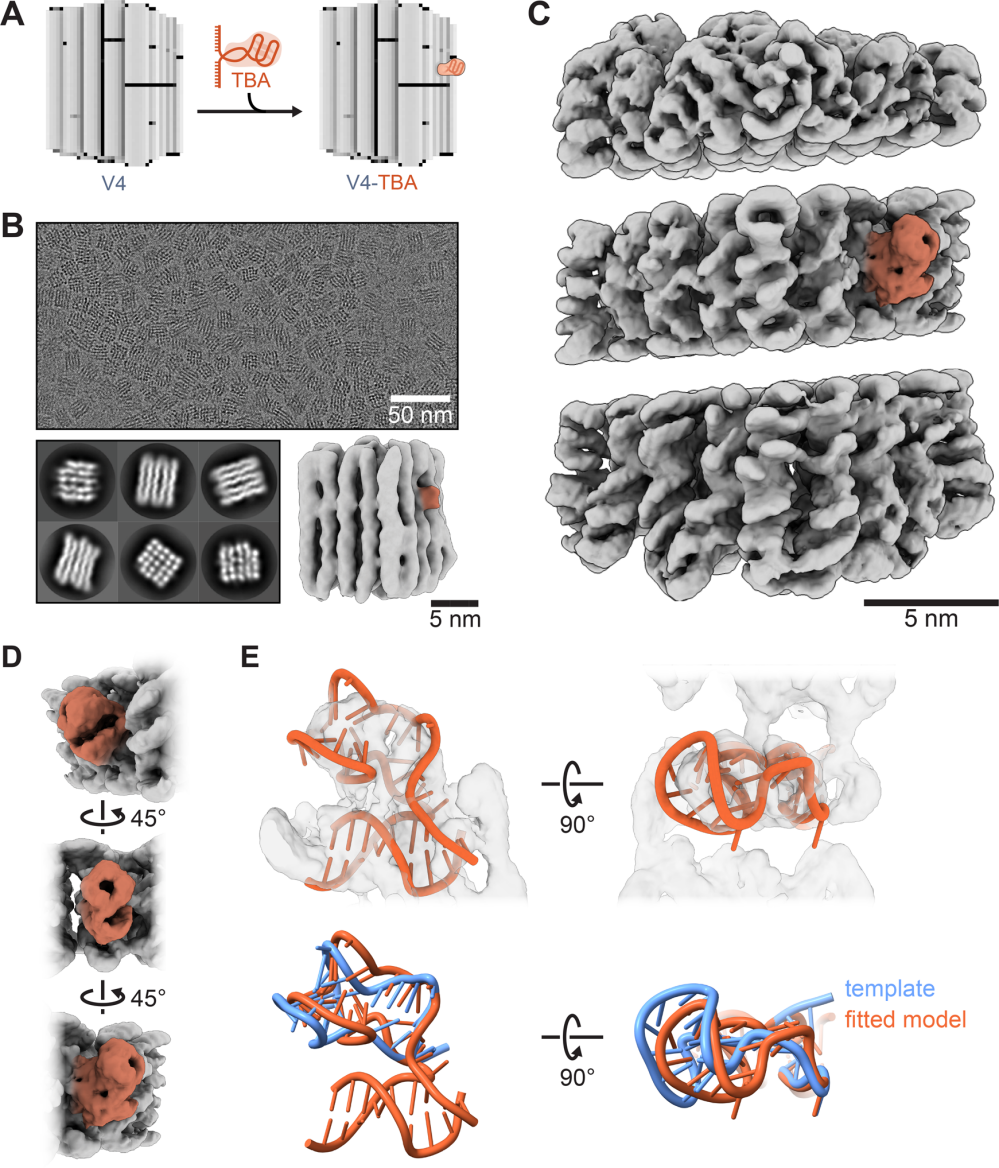
Incorporation of a thrombin binding DNA aptamer into DNA origami and its cryo-EM single-particle analysis. A) Schematic depiction of the DNA origami structure with the DNA thrombin binding aptamer connected to it. B) Cryo-EM micrograph of the specimen alongside its 2D classification outcomes, accompanied by a 3D reconstruction of the DNA origami complex bearing the affixed aptamer. C) Segregated representations of the supple peripheral segments and the more inflexible central components acquired via multibody analysis. The DNA aptamer is accentuated with distinct colors for enhanced visualization. D) Showcasing the DNA aptamer in color-coded form, viewable from multiple vantage points, to provide a comprehensive visual assessment. E) The upper panel model of the aptamer-scaffold DNA fusion fitted into the cryo-EM map. The fitted model is compared to the original template extracted from the protein-bound crystal structure (PDB entry 4I7Y) in the lower panel.

We note that we could not discern individual base pairs in our map, which is required for de novo structure determination of the aptamer (we used a reference crystal structure). The required resolution could not be reached in the current study, which we attribute to the residual intrinsic heterogeneity of the DNA origami host structure, as caused by interhelical breathing motions and defects, and to the flexibility of the connections between the guest molecule and DNA origami support. To enhance the resolution of DNA origami objects and potential guest molecules in future studies, it is worth exploring additional strategies that could minimize these flexibility issues. One solution could be stabilizing the DNA origami structure by cross-linking or including rigidifying elements. The attachment points between the guest molecule and origami could be rigidified by using modified nucleotides or intercalating agents that reduce flexibility. Furthermore, steric constraints could be introduced that limit the conformational freedom of the guest molecules, for example, by attaching them in a cavity. Advances in cryo-EM technology and image processing algorithms may also contribute to improved resolution.

In summary, we investigated how different modifications affect the folding of DNA origami structures. These modifications included adjusting the number of scaffold crossovers, varying staple length ranges, and using purer staples to reach improved assembly outcomes, as measured by electrophoretic mobility and cryo-EM SPA under otherwise consistent conditions. Additionally, using purer staples further improved the outcomes. Finally, we successfully incorporated a small 27-base-long DNA aptamer into a DNA origami scaffold and resolved its overall shape using cryo-EM. Overall, our findings contribute to advancing the design and optimization of DNA origami for various applications in DNA nanotechnology, allowing researchers to make more informed decisions about the design and use of these structures.

## DNA Origami Design, Folding, and Purification

The fabrication of DNA origami structures was carried out using caDNAno sq v0.1 and caDNAno v2 software.^6^ These structures underwent folding in standard “folding buffers” known as FoBx, which consisted of *x* mM MgCl_2_, 5 mM Tris base, 1 mM EDTA, and 5 mM NaCl at pH 8. Thermal annealing was conducted using Tetrad thermal cycling devices, applying the appropriate thermal annealing ramps. Detailed folding conditions for each specific origami structure can be found in the Supporting Information, Supplementary Tables. Staple strands were obtained from Integrated DNA Technologies with standard desalting or HPLC purification. The origami scaffold and staple routing were visually depicted in the Supporting Information, Supplementary Figures. Size exclusion chromatography, PEG precipitation, and amicon filters were utilized to purify the origami structures.

## Gel Electrophoresis

Agarose gels with either a 2 or 4% concentration were employed to evaluate the quality of DNA origami folding (Supporting Information, Supplementary Figures 5–9). These gels were prepared using 0.5 × TBE buffer (22 mM tris base, 22 mM boric acid, and 0.5 mM EDTA) supplemented with 5.5 mM MgCl_2_. Electrophoresis was carried out in the same buffer solution at a voltage of 90 V for 2 to 4 h. The gels were cooled in water or an ice bath to maintain optimal conditions. Subsequently, the gels were scanned using a Typhoon FLA 9500 laser scanner (GE Healthcare) with a 50 μm/pixel resolution. The binding targets were labeled to facilitate the assessment of DNA origami structure folding quality.

## Preparation of Vitrified Specimens

Quantifoil 200-mesh copper grids with R1.2/1.3 holey carbon support films were utilized to prepare the cryo-EM grids. The grids were treated with an EMS K100X plasma cleaner (Electron Microscopy Sciences) for 90 s, undergoing a glow discharge process in a high-pressure air environment. The sample was carefully applied to the grid within the Vitrobot Mark IV chamber (FEI). Before sample application, the chamber conditions were adjusted to maintain 100% humidity at a temperature of 4 °C. Once the sample was applied, the excess solution was blotted for 3 s using a blot force of 20. Immediately after blotting, the grid was plunged into liquid ethane, rapidly freezing the sample and ensuring vitrification for subsequent cryo-EM analysis.

## Data Acquisition

The data were obtained by using a Titan Krios microscope (ThermoFisher Scientific) operating at 300 kV. The microscope had a Falcon3 direct electron detector and a CS corrector. Movies were recorded in nanoprobe mode employing a 50 μm C2 and a 100 μm objective aperture, with data sets for V2, V4-SD, V4-HPLC, and V4-Aptamer captured at a magnified pixel size of 0.86 Å. In contrast, the V1 and V3 data sets were acquired at a pixel size of 1.4 Å (Supporting Information, Supplementary Table 1). Each movie was acquired with a total dose of 50 e^–^/Å^2^. The data collection process was automated by using EPU software (ThermoFisher Scientific).

## Data Processing

The collected movies underwent motion correction using RELION’s implementation of a MotionCor2 algorithm.^38^ CTF estimation was then carried out using CTFFIND-4.1 software on the non-dose-weighted micrographs.^39^ For particle selection, either the crYOLO software^40^ or a Laplacian-of-Gaussian automated picking routine on the dose-weighted micrographs in Relion4 was employed. Initial models were generated in Relion using the extracted data. Multibody refinement analysis was conducted in Relion, where consensus maps were segmented into specific regions using the crop function in UCSF Chimera.^41^ These segments were then low-pass filtered, converted to binary form, and appended with soft-edge voxels to construct the masks required for multibody refinement. The employed masks were tight, encompassing all of the essential features in the consensus map. Following multibody refinement, the maps underwent postprocessing with wide, low-pass-filtered masks, the same as those applied in the consensus map postprocessing to calculate the Fourier shell correlations (FSCs) (Supporting Information, Supplementary Figure 16). Furthermore, the final maps had enhanced consistency using the deepEMhancer and LocSpiral software packages, resulting in sharpened and improved representations.^42,43^

## Model Fitting and Refinement

We used the coordinates of the previously determined crystal structure of the aptamer DNA strand (Protein Data Bank entry 4I7Y) as a template to rigid-body fit an atomic model into the density using COOT.^44^ To interpret the connection between the aptamer and the adjacent DNA scaffold, we generated a 16-mer canonical B-form DNA helix in the COOT and conducted rigid body fitting. The bases were renumbered according to the aptamer sequence from PDB entry 4I7Y, and DNA strands were joined manually in COOT. Flexible fitting was performed using CHIMERAX/ISOLDE^45,46^ in combination with individual self-distance restraints for the aptamer segment and the scaffold DNA helix. Next, the model underwent real-space refinement using the FastRelax protocol within ROSETTA.^47^ This refinement process incorporates density scoring and torsional reference model restraints. The reference model restraints were generated using phenix.real_space_refine, utilizing nucleotides 5–20 from 4I7Y (excluding nucleotides 8 and 9 in chain A) and the ideal DNA template (excluding nucleotides 8 and 38 in chain B) (Figure 3E).^48^

## Data Availability

All maps and the fitted model that support the findings of this study are available in the EMDB^49^ and in the Protein Data Bank (PDB),^50^ respectively: EMD-19767, EMD-19769, EMD-19770, EMD-19775, EMD-19776, EMD-19867, EMD-19874, EMD-19875, EMD-19876, and 9EOQ.

## Supporting Information Available

DNA origami design files, details of folding validation and purification methods as confirmed by gel electrophoresis, specifics of the cryo-EM processes, and complete sequences of the scaffolds employed in our experiments (PDF)
