# Supplementary material for: Designing Rigid DNA Origami Templates for Molecular Visualization Using Cryo-EM
Source: Nano Lett. 2024 Apr 11;24(16):5031–8. doi: 10.1021/acs.nanolett.4c00915 (PMC11057029; doi:10.1021/acs.nanolett.4c00915)
Supplement: Supplementary file 1 — nl4c00915_si_001.pdf [file nl4c00915_si_001.pdf]

# Supplementary Information:

# Designing Rigid DNA Origami Templates for Molecular Visualization using Cryo-EM

Ali Khoshouei[1,2], Georg Kempf[3], Volodymyr Mykhailiuk[1,2], Johanna Mariko Griessing[1,2], Maximilian Nicolas Honemann[1,2], Lukas Kater[3], Simone Cavadini[3], and Hendrik Dietz*[1,2]

[1] Laboratory for Biomolecular Nanotechnology, Department of Biosciences, School of Natural Sciences, Technical University of Munich, Am Coulombwall 4a, 85748 Garching, Germany.
[2] Munich Institute of Biomedical Engineering, Technical University of Munich, Boltzmannstraße 11, 85748 Garching, Germany.
[3] Friedrich Miescher Institute for Biomedical Research, Maulbeerstrasse 66, 4058 Basel, Switzerland

*Corresponding author: dietz@tum.de

**Supplementary information**

**Content**

**Supplementary Figures 1 – 16**

**Supplementary Table 1**

**Supplementary Notes 1 – 2**

## Supplementary Figures

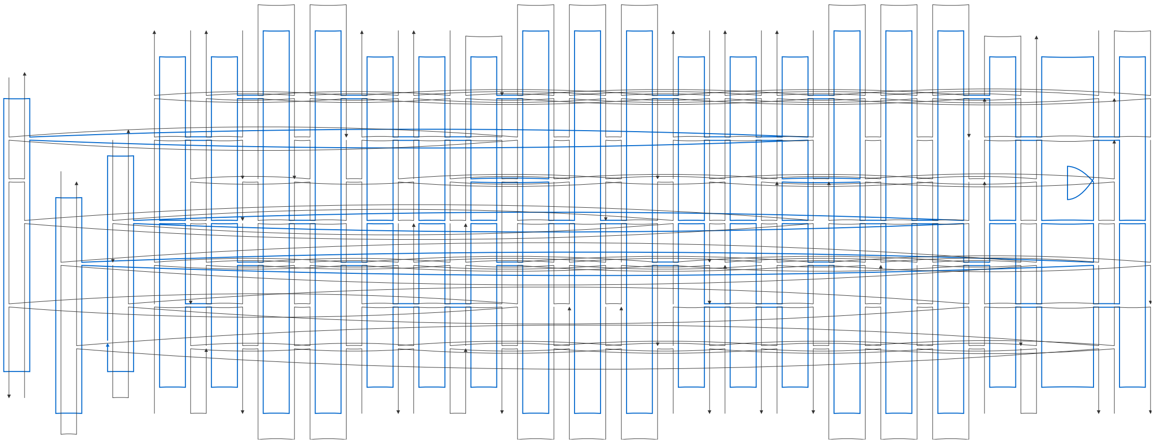

**Supplementary Figure 1 | Design diagram of the "V1" object.** The design diagram of the V1 was designed using the Cadnano software. The scaffold size is 2873 nucleotides of single-stranded DNA.

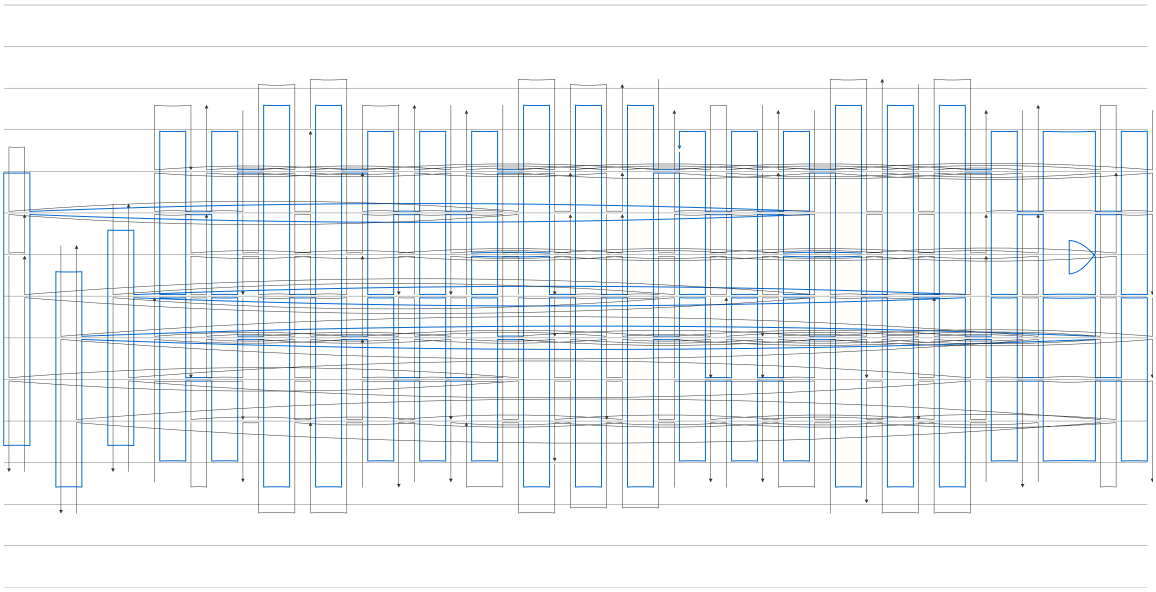

**Supplementary Figure 2 | Design diagram of the "V2" object.** The design diagram of the V2 was designed using the Cadnano software. The scaffold size is 2873 nucleotides of single-stranded DNA.

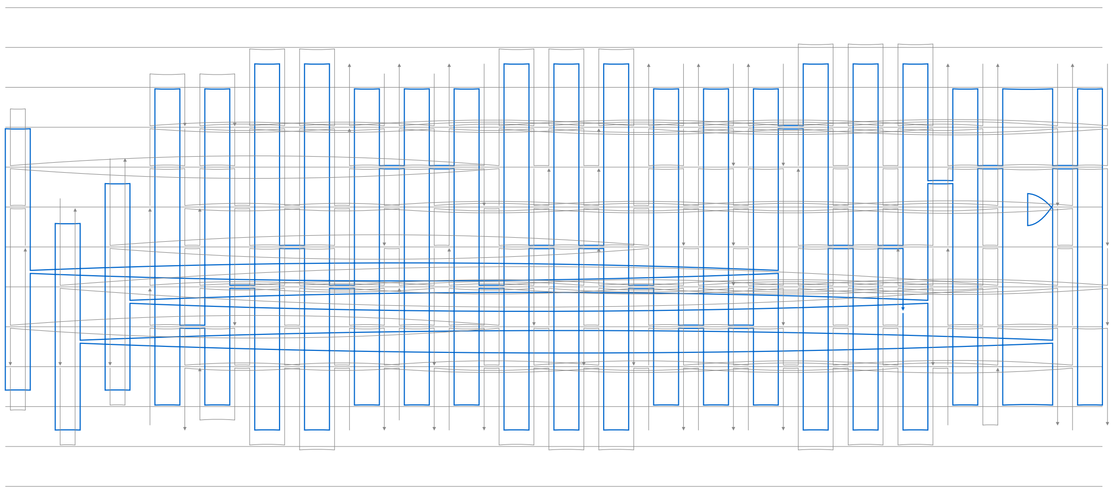

**Supplementary Figure 3 | Design diagram of the "V3" object.** The design diagram of the V3 was designed using the Cadnano software. The scaffold size is 2873 nucleotides of single-stranded DNA.

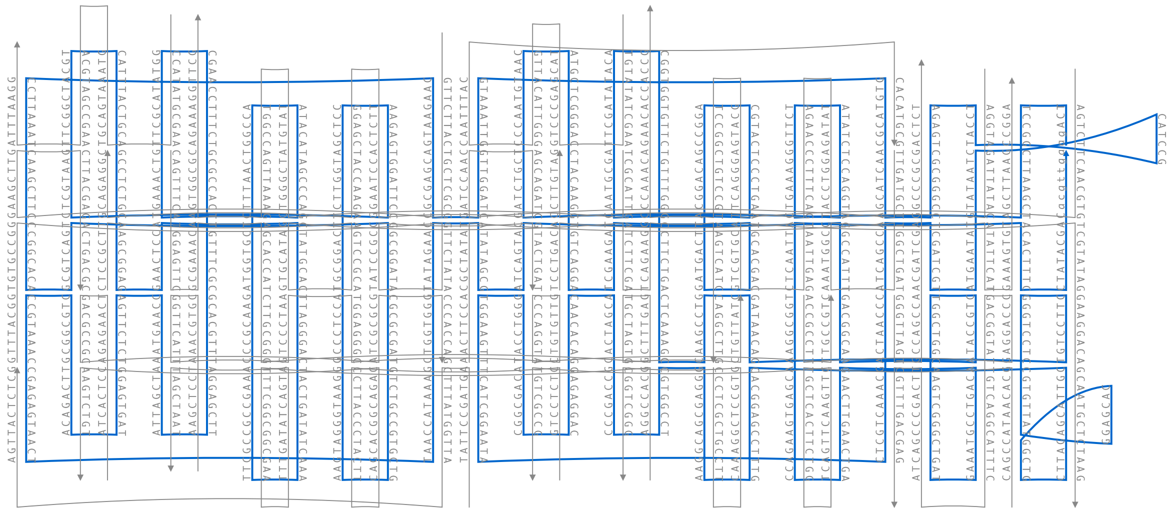

**Supplementary Figure 4 | Design diagram of the "V4" object.** The design diagram of the V4 was designed using the Cadnano software. The scaffold size is 1033 nucleotides of single-stranded DNA.

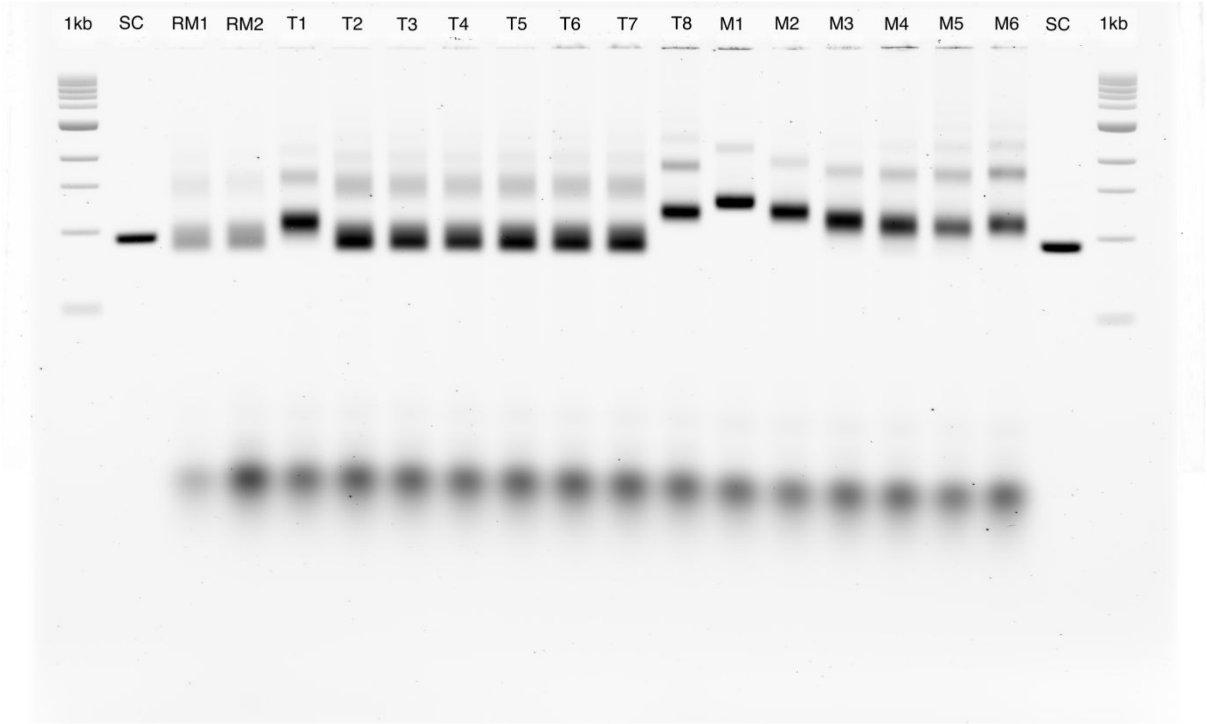


**Supplementary Figure 5 | Folding screen of the V1.** Fluorescent gel electrophoresis image of a 2% agarose gel containing 5.5 mM $MgCl_2$, run at 90 V for 120 minutes. The gel exhibits the following loaded samples: 1kb = 1kb ladder, sc = 2873 nt long scaffold strand, RM1 = folding interval: 60 - 44 °C, 20 mM $MgCl_2$, 1:4 scaffold to staple ratio, RM2 = folding interval: 60 - 44 °C, 20 mM $MgCl_2$, 1:10 scaffold to staple ratio, temperature screen T1 - T8 = 20 mM $MgCl_2$ (T1 = folding interval: 50 - 47 °C, T2 = folding interval: 52 - 49 °C, T3 = folding interval: 54 - 51 °C, T4 = folding interval: 56 - 53 °C, T5 = folding interval: 58 - 55 °C, T6 = folding interval: 60 - 57 °C, T7 = folding interval: 62 - 59 °C, T8 = folding interval: 64 - 61 °C), magnesium screen M5 - M30 = folding interval: 60 - 44 °C (M5 = 5 mM $MgCl_2$, M10 = 10 mM $MgCl_2$, M15 = 15 mM $MgCl_2$, M20 = 20 mM $MgCl_2$, M25 = 25 mM $MgCl_2$, M30 = 30 mM $MgCl_2$). The T4 condition was selected for further optimization and folding of the cryo-EM sample.

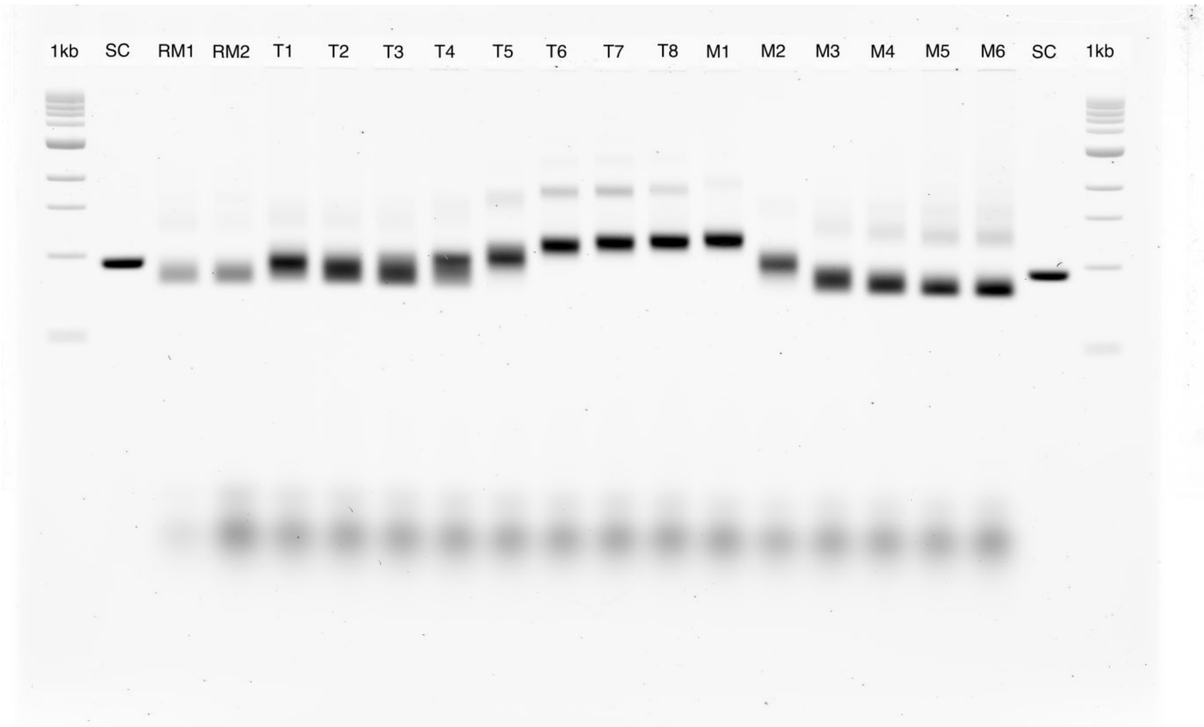


**Supplementary Figure 6 | Folding screen of the V2.** Fluorescent gel electrophoresis image of a 2% agarose gel containing 5.5 mM $MgCl_2$, run at 90 V for 120 minutes. The gel exhibits the following loaded samples: 1kb = 1kb ladder, sc = 2873 nt long scaffold strand, RM1 = folding interval: 60 - 44 °C, 20 mM $MgCl_2$, 1:4 scaffold to staple ratio, RM2 = folding interval: 60 - 44 °C, 20 mM $MgCl_2$, 1:10 scaffold to staple ratio, temperature screen T1 - T8 = 20 mM $MgCl_2$ (T1 = folding interval: 50 - 47 °C, T2 = folding interval: 52 - 49 °C, T3 = folding interval: 54 - 51 °C, T4 = folding interval: 56 - 53 °C, T5 = folding interval: 58 - 55 °C, T6 = folding interval: 60 - 57 °C, T7 = folding interval: 62 - 59 °C, T8 = folding interval: 64 - 61 °C), magnesium screen M5 - M30 = folding interval: 60 - 44 °C (M5 = 5 mM $MgCl_2$, M10 = 10 mM $MgCl_2$, M15 = 15 mM $MgCl_2$, M20 = 20 mM $MgCl_2$, M25 = 25 mM $MgCl_2$, M30 = 30 mM $MgCl_2$). The M6 condition was selected for further optimization and folding of the cryo-EM sample.

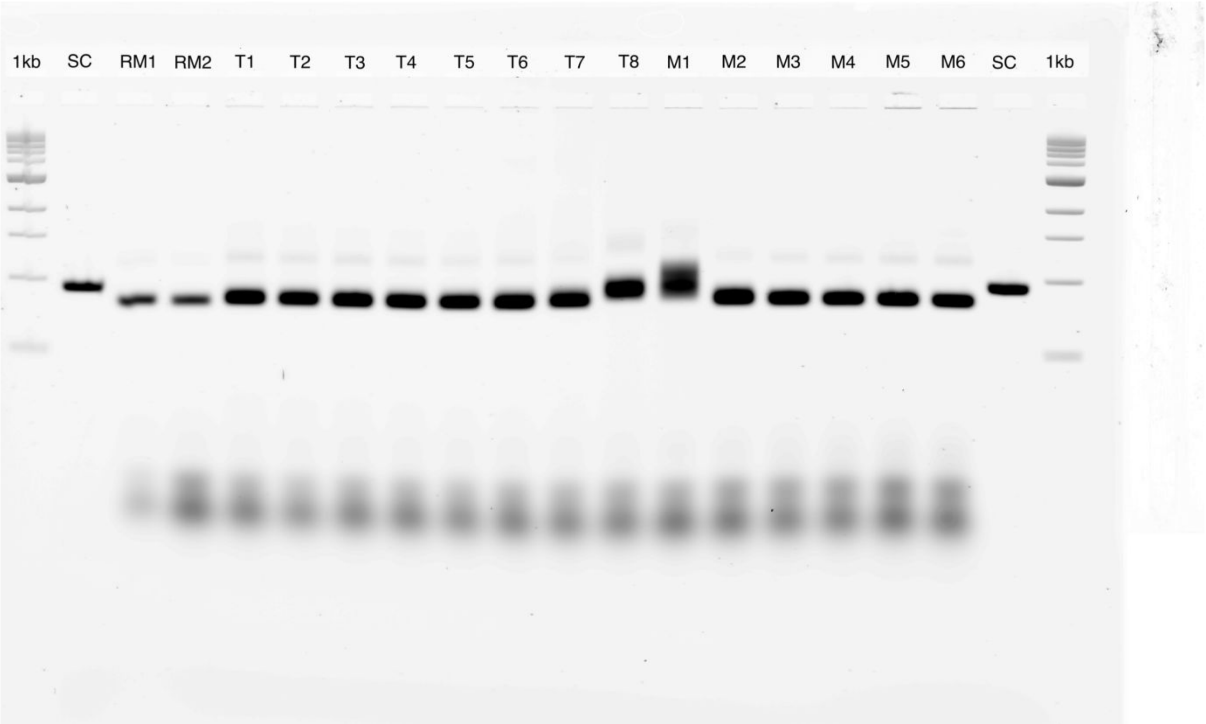


**Supplementary Figure 7 | Folding screen of the V3.** Fluorescent gel electrophoresis image of a 2% agarose gel containing 5.5 mM $MgCl_2$, run at 90 V for 120 minutes. The gel exhibits the following loaded samples: 1kb = 1kb ladder, sc = 2873 nt long scaffold strand, RM1 = folding interval: 60 - 44 °C, 20 mM $MgCl_2$, 1:4 scaffold to staple ratio, RM2 = folding interval: 60 - 44 °C, 20 mM $MgCl_2$, 1:10 scaffold to staple ratio, temperature screen T1 - T8 = 20 mM $MgCl_2$ (T1 = folding interval: 50 - 47 °C, T2 = folding interval: 52 - 49 °C, T3 = folding interval: 54 - 51 °C, T4 = folding interval: 56 - 53 °C, T5 = folding interval: 58 - 55 °C, T6 = folding interval: 60 - 57 °C, T7 = folding interval: 62 - 59 °C, T8 = folding interval: 64 - 61 °C), magnesium screen M5 - M30 = folding interval: 60 - 44 °C (M5 = 5 mM $MgCl_2$, M10 = 10 mM $MgCl_2$, M15 = 15 mM $MgCl_2$, M20 = 20 mM $MgCl_2$, M25 = 25 mM $MgCl_2$, M30 = 30 mM $MgCl_2$). The T4 condition was selected for further optimization and folding of the cryo-EM sample.

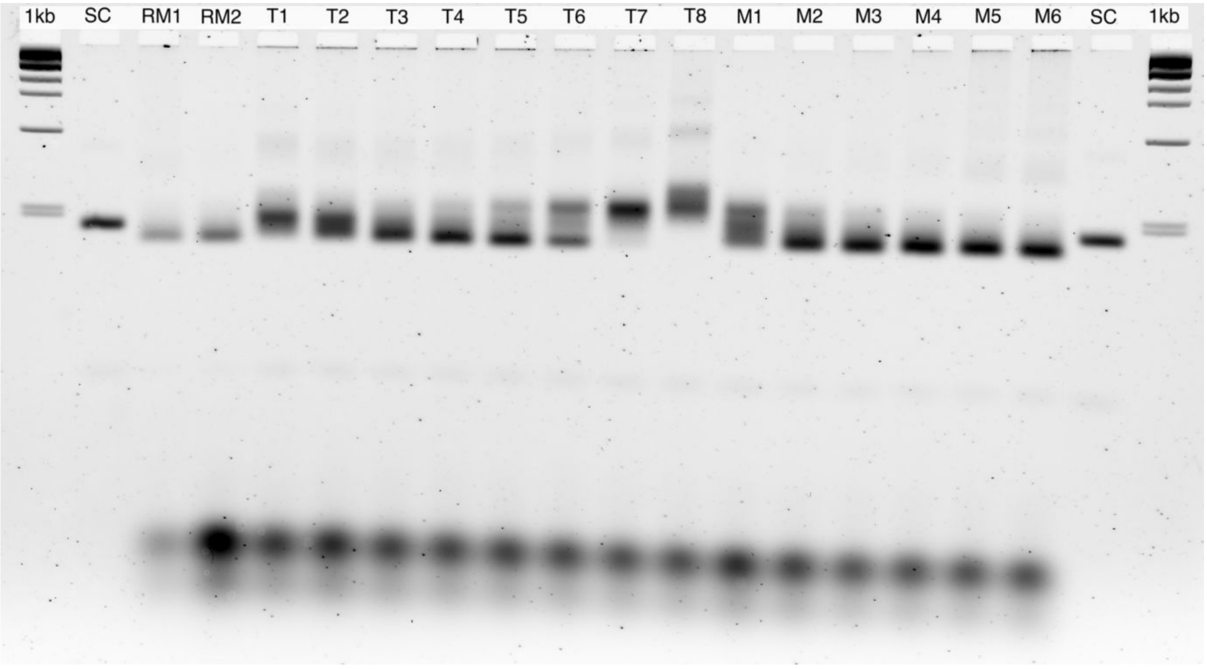


**Supplementary Figure 8 | Folding screen of the V4.** Fluorescent gel electrophoresis image of a 4% agarose gel containing 5.5 mM $MgCl_2$, run at 90 V for 120 minutes. The gel exhibits the following loaded samples: 1kb = 1kb ladder, sc = 1033 nt long scaffold strand, RM1 = folding interval: 60 - 44 °C, 20 mM $MgCl_2$, 1:4 scaffold to staple ratio, RM2 = folding interval: 60 - 44 °C, 20 mM $MgCl_2$, 1:10 scaffold to staple ratio, temperature screen T1 - T8 = 20 mM $MgCl_2$ (T1 = folding interval: 50 - 47 °C, T2 = folding interval: 52 - 49 °C, T3 = folding interval: 54 - 51 °C, T4 = folding interval: 56 - 53 °C, T5 = folding interval: 58 - 55 °C, T6 = folding interval: 60 - 57 °C, T7 = folding interval: 62 - 59 °C, T8 = folding interval: 64 - 61 °C), magnesium screen M5 - M30 = folding interval: 60 - 44 °C (M1 = 10 mM $MgCl_2$, M2 = 15 mM $MgCl_2$, M3 = 20 mM $MgCl_2$, M4 = 25 mM $MgCl_2$, M5 = 30 mM $MgCl_2$, M6 = 35 mM $MgCl_2$). The M6 condition was selected for further optimization and folding of the cryo-EM sample.

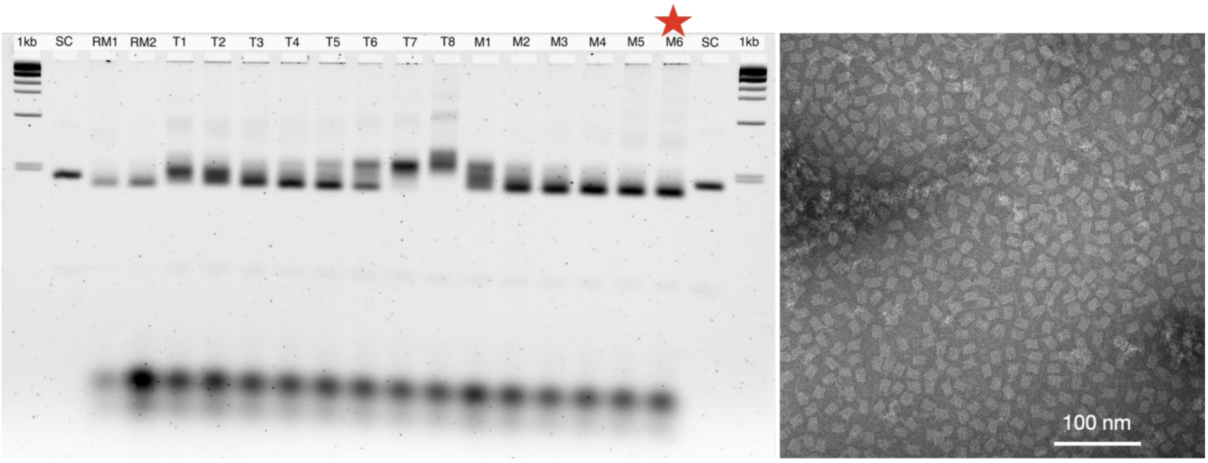


**Supplementary Figure 9 | Confirmation of folding quality through negative stain transmission electron microscopy (TEM) analysis.** The folding screen of V4 from the M6 lane reveals the presence of the folded origami structure. The folding process was performed at a magnesium chloride ($MgCl_2$) concentration of 35 mM and temperature intervals ranging from 60 to 44°C.

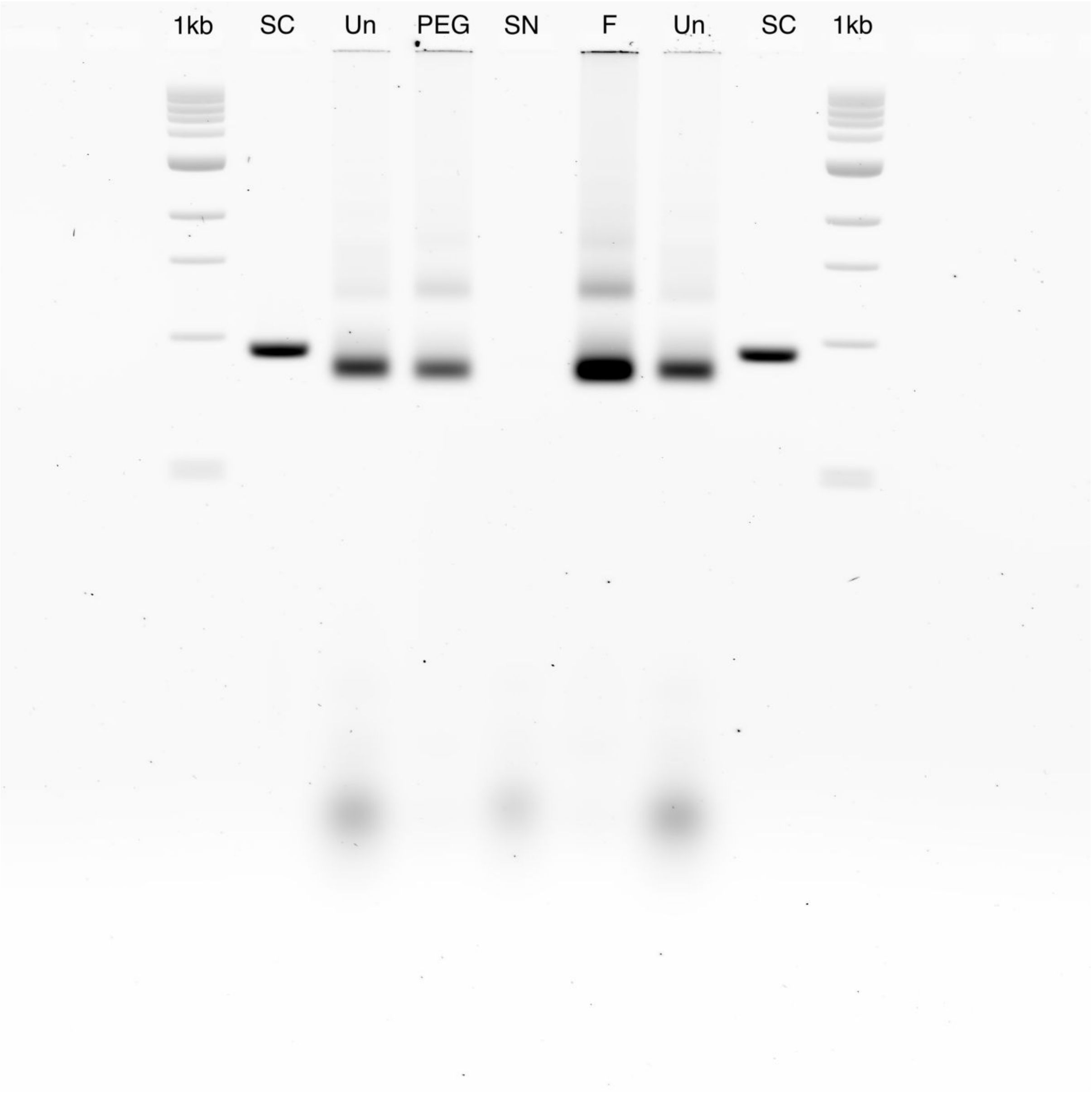


**Supplementary Figure 10 | Purification steps employed for the purification of V2.** Gel electrophoresis data displays the following lanes: 1kb - 1kb ladder, SC - 2873 nucleotides DNA Scaffold, Un - unpurified folded DNA origami, PEG - PEG-precipitated purified sample, SN - supernatants from PEG purification containing excess staples only, F - Amicon filter utilized for additional concentration and sample preparation for cryo-EM experiments.

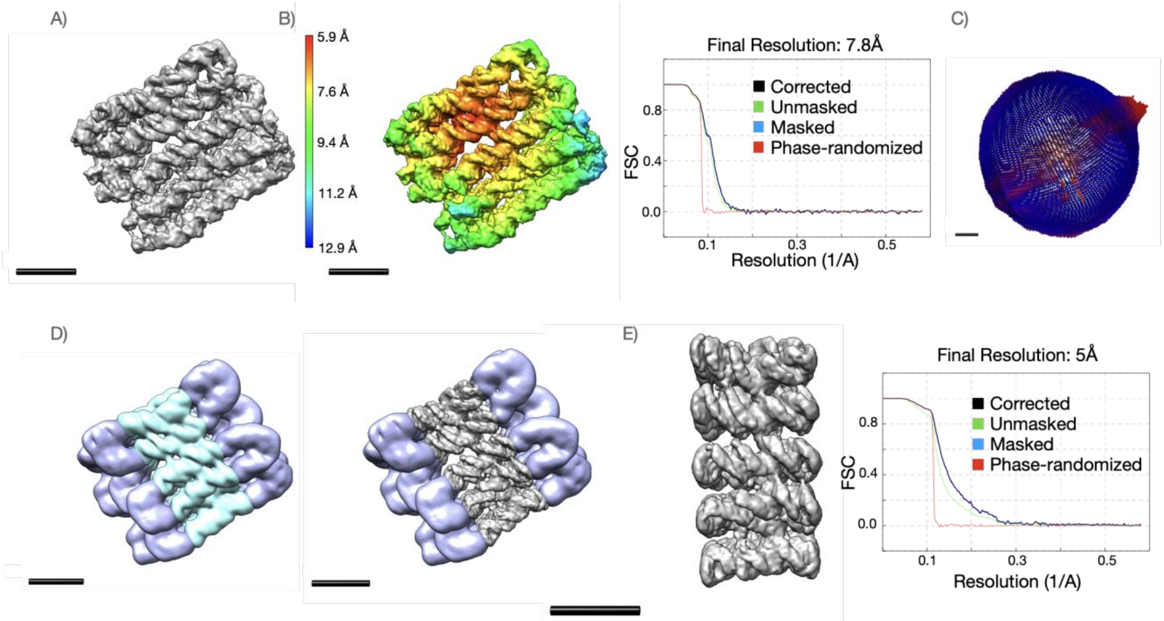


**Supplementary Figure 11: Cryo-EM Structural Analysis of DNA Origami V4 Utilizing Desalted Purified Staples:** A) Refined 3D cryo-EM structure of the DNA origami V4, obtained using desalted purified staple. B) Color-coded local resolution map, with an overall resolution of 7.8 Å. C) Distribution of angular orientations. D) Multi-body analysis employed to enhance resolution in various structural regions. The central portion corresponds to a less flexible region and is treated as an independent entity. The mask employed for the multi-body analysis is precise, encompassing all pertinent features without omitting critical data. E) The rigid segment of the DNA origami underwent refinement using multi-body analysis. The same mask employed for the consensus map indicates resolution, yielding an overall resolution of 5 Å. Scale bar: 5 nm

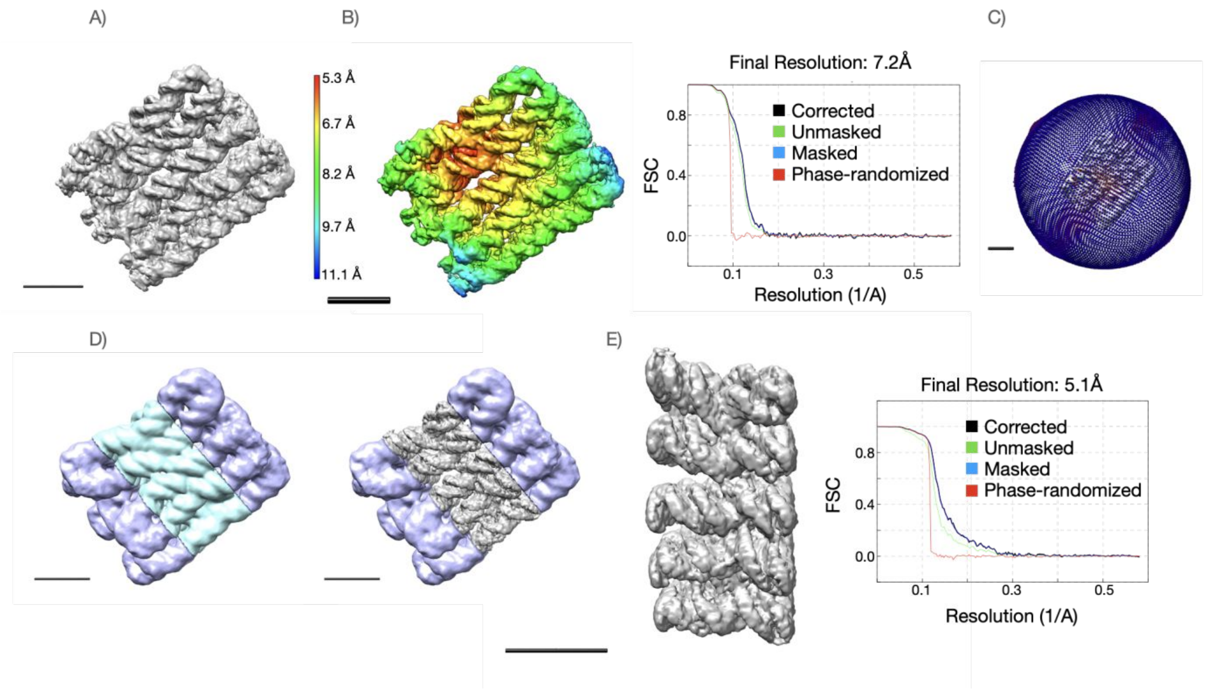


**Supplementary Figure 12: Cryo-EM Structural Analysis of DNA Origami V4 Utilizing HPLC Purified Staples:** A) Refined 3D cryo-EM structure of the DNA origami V4, obtained using HPLC purified staple. B) Color-coded local resolution map, with an overall resolution of 7.2 Å. C) Distribution of angular orientations. D) Multi-body analysis employed to enhance resolution in various structural regions. The central portion corresponds to a less flexible region and is treated as an independent entity. The mask employed for the multi-body analysis is precise, encompassing all pertinent features without omitting critical data. E) The rigid segment of the DNA origami underwent refinement using multi-body analysis. The same mask employed for the consensus map is used to indicate resolution, yielding an overall resolution of 5.1 Å. Scale bar: 5 nm

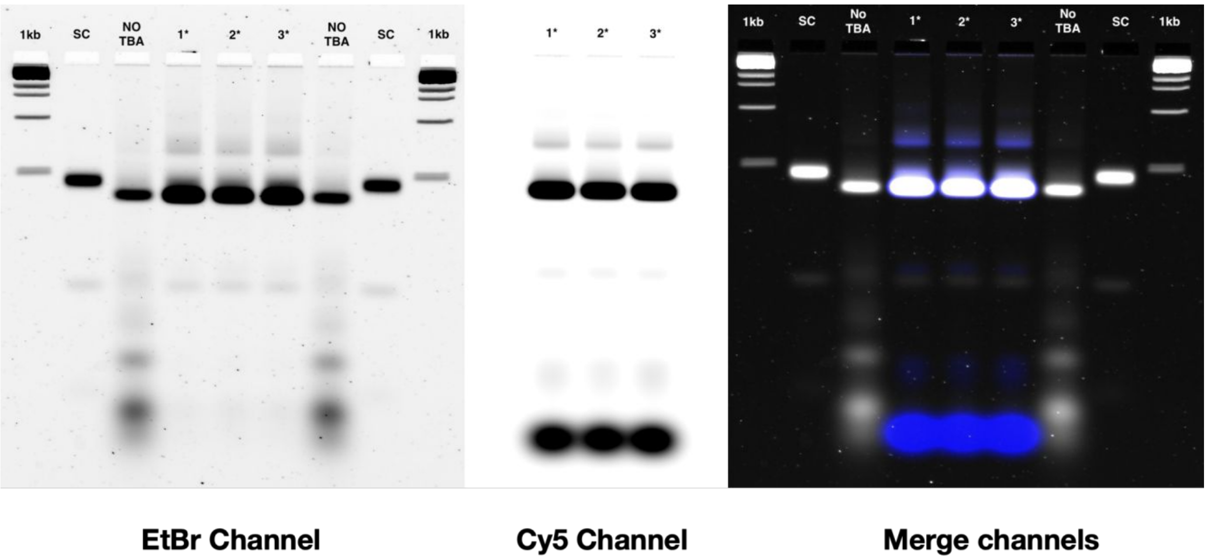


**Supplementary Figure 13 | Assessment of the binding of Cy5-labeled TBA to V4.** In the gel on the left side: 1) The origami and TBA are folded together and subsequently cooled to 4°C. 2) TBA is added after folding and left at room temperature overnight. 3) The origami is folded first, followed by the addition of TBA, and then cooled and maintained at 4°C. In the middle-scanned gel, conducted in the Cy5 channel, signals are exclusively detected in the lanes containing TBA. On the right, the merged image combines data from the EtBr channel with the Cy5 channel, enhancing the visualization of TBA binding.

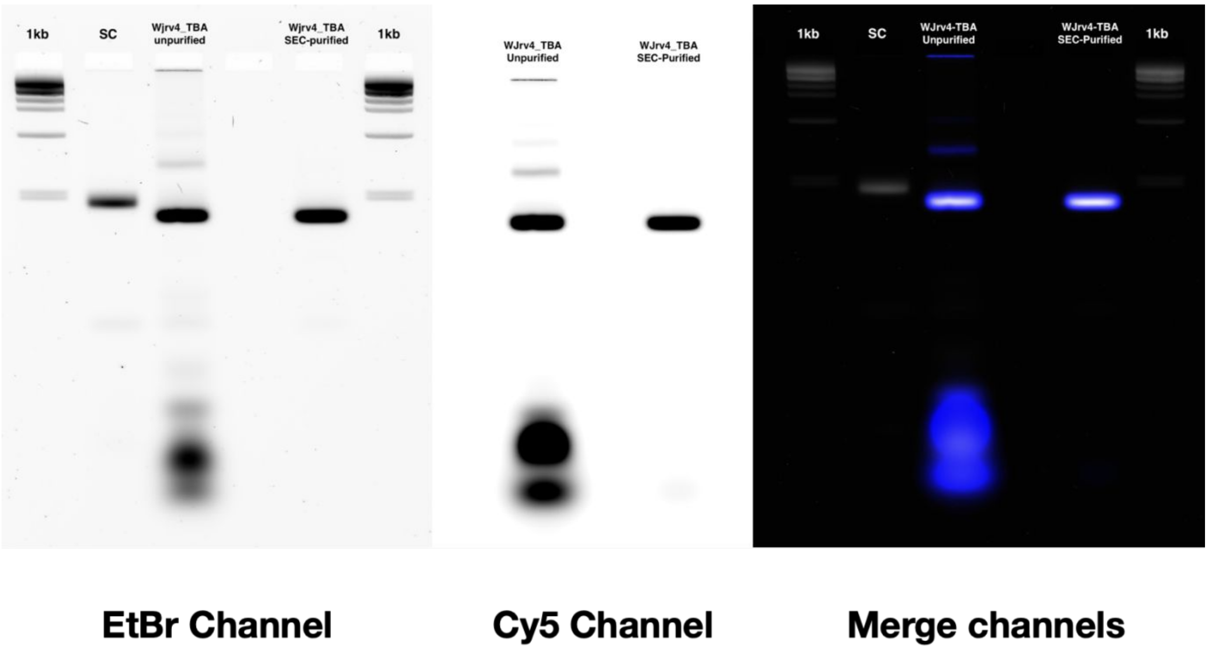


**Supplementary Figure 14 | Purification workflow for DNA origami V4_TBA.** The purification process involves size exclusion chromatography (SEC). On the left, a gel electrophoresis scan is presented, showcasing the following lanes from left to right: 1kb ladder, scaffold, unpurified sample, and SEC-purified sample concentrated using amicon filters. The middle panel displays the cy5 channels, confirming the presence of the thrombin-binding aptamer (TBA) after purification. The right panel shows the merged channel, providing a comprehensive view of the purified sample.

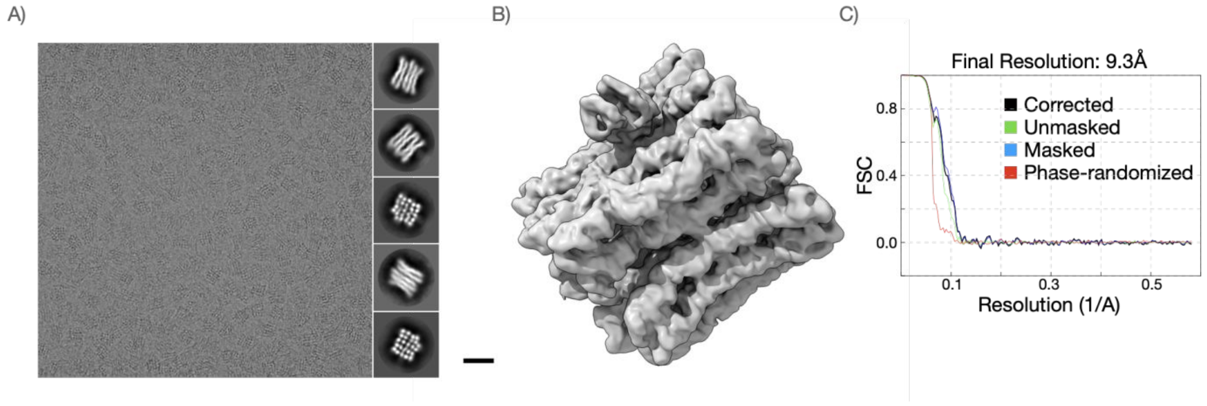


**Supplementary Figure 15 | Investigating the interaction between origami V4_TBA and Thrombin and evaluating the functionality of the DNA aptamer when bound to the DNA origami.** A) A representative cryo-EM micrograph and 2D class averages derived from all micrographs. B) Multibody reconstructions illustrate the DNA origami's rigid and flexible sections. C) Evaluation of the overall resolution in the central region where TBA and Thrombin are present. Scale bar: 2 nm

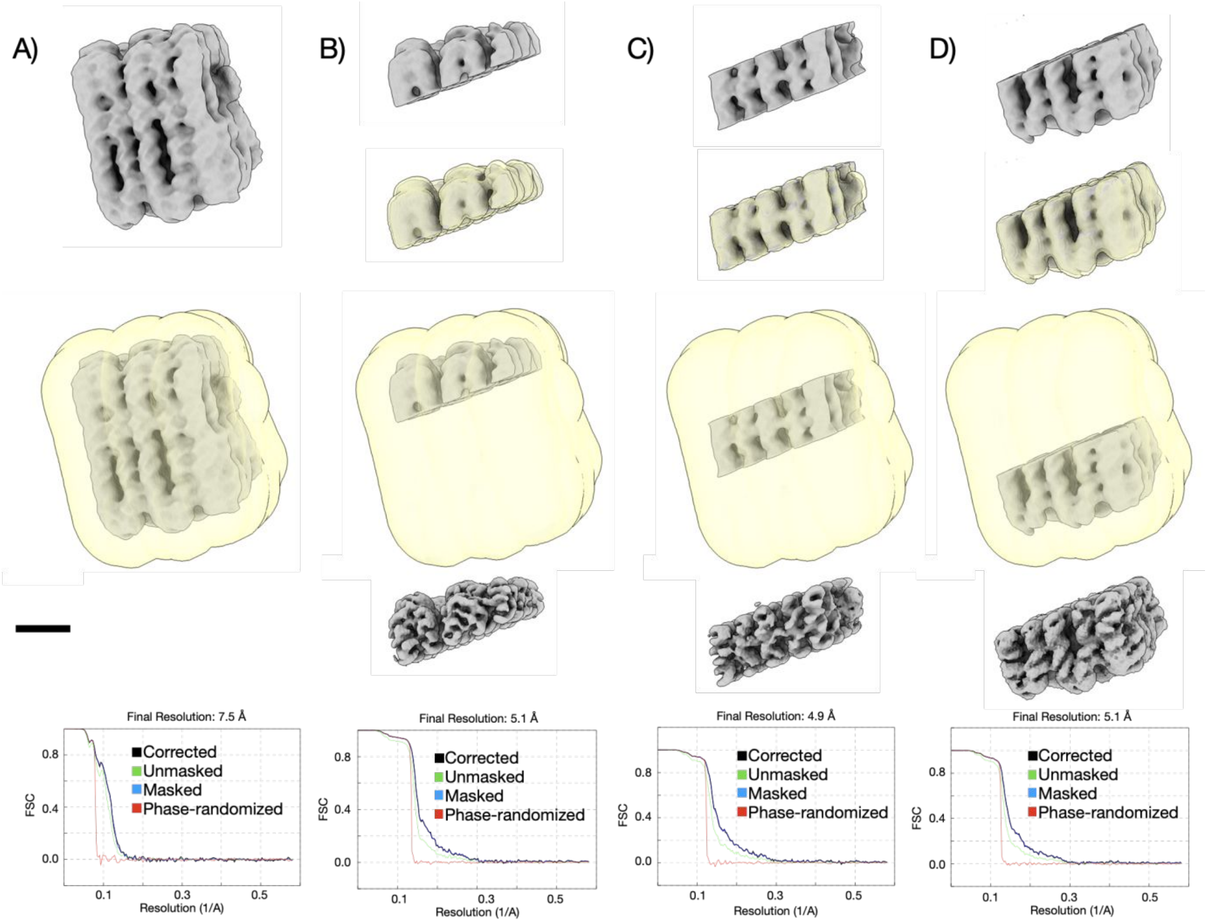


**Supplementary Figure 16 | Data Processing Workflow:** A) From top to bottom: the 3D refined cryo-EM map of the DNA origami V4-Aptamer complex, the resolution-determining mask, and the FSC curve, with a final resolution of 7.5Å. B) In sequence: the upper segment of the 3D refined structure, the tight mask for multibody analysis, the resolution-specifying mask, the sharpened map, and the FSC curve displaying a final resolution of 5.1Å. C) Top to bottom: the middle segment of the 3D refined structure containing the attached aptamer, the tight mask for multibody analysis, the final resolution mask, the sharpened map, and the FSC curve indicating a final resolution of 4.9 Å. D) From top to bottom: the lower segment of the 3D refined structure, the tight mask for multibody analysis, the mask determining final resolution, the sharpened map, and the FSC curve showing a final resolution of 5.1Å. Scale bar: 5 nm.

# Supplementary Table

**Supplementary Table 1 | Cryo-EM grid preparation and imaging conditions.**

| Object | DNA origami Concentration (µM) | Grid Type | # of particles for refinement | # of fractions | Dose (e/A^2) | Pixel size (A/pix) | Resolution of resulting 3D map (A) |
|---|---|---|---|---|---|---|---|
| **V1** | 3 | Quantifoil 1.2/1.3-200 mesh | 140317 | 13 | 51 | 1.4 | 8.6 |
| **V2** | 6.75 | Quantifoil 1.2/1.3-200 mesh | 151581 | 20 | 50 | 0.86 | 7.5 |
| **V3** | 8 | Quantifoil 1.2/1.3-Au, 300 mesh | 295463 | 10 | 50 | 1.4 | 8.3 |
| **V4_SD** | 14.1 | Quantifoil -1.2/1.3-200 mesh | 408927 | 20 | 50 | 0.86 | 7.8 |
| **V4_HPLC** | 10.3 | Quantifoil -1.2/1.3-200 mesh | 389297 | 20 | 50 | 0.86 | 7.2 |
| **V4_TBA** | 10 | Quantifoil -1.2/1.3-200 mesh | 966383 | 20 | 50 | 0.86 | 7.5 |
| **V4_TBA_Thrombin** | 10.5 | Quantifoil -1.2/1.3-200 mesh | 133161 | 20 | 40 | 0.86 | 9.3 |

## Supplementary Notes

**Note 1: 2873 scaffold:**

GTGGCACTTTTCGGGGAAATGTGCGCGGAACCCCTATTTGTTTATTTTTCTAAATACATTC
AAATATGTATCCGCTCATGAGACAATAACCCTGATAAATGCTTCAATAATATTGAAAAAG
GAAGAGTATGAGTATTCAACATTTCCGTGTCGCCCTTATTCCCTTTTTTGCGGCATTTTGC
CTTCCTGTTTTTGCTCACCCAGAAACGCTGGTGAAAGTAAAAGATGCTGAAGATCAGTTG
GGTGCACGAGTGGGTTACATCGAACTGGATCTCAACAGCGGTAAGATCCTTGAGAGTTTT
CGCCCCGAAGAACGTTTTCCAATGATGAGCACTTTTAAAGTTCTGCTATGTGGCGCGGTA
TTATCCCGTATTGACGCCGGGCAAGAGCAACTCGGTCGCCGCATACACTATTCTCAGAAT
GACTTGGTTGAGTACTCACCAGTCACAGAAAAGCATCTTACGGATGGCATGACAGTAAG
AGAATTATGCAGTGCTGCCATAACCATGAGTGATAACACTGCGGCCAACTTACTTCTGAC
AACGATCGGAGGACCGAAGGAGCTAACCGCTTTTTTGCACAACATGGGGGATCATGTAA
CTCGCCTTGATCGTTGGGAACCGGAGCTGAATGAAGCCATACCAAACGACGAGCGTGAC
ACCACGATGCCTGTAGCAATGGCAACAACGTTGCGCAAACTATTAACTGGCGAACTACTT
ACTCTAGCTTCCCGGCAACAATTAATAGACTGGATGGAGGCGGATAAAGTTGCAGGACC
ACTTCTGCGCTCGGCCCTTCCGGCTGGCTGGTTTATTGCTGATAAATCTGGAGCCGGTGA
GCGTGGGTCACGCGGTATCATTGCAGCACTGGGGCCAGATGGTAAGCCCTCCCGTATCGT
AGTTATCTACACGACGGGGAGTCAGGCAACTATGGATGAACGAAATAGACAGATCGCTG
AGATAGGTGCCTCACTGATTAAGCATTGGTAACTGTCAGACCAAGTTTACTCATATATAC
TTTAGATTGATTTAAAACTTCATTTTTAATTTAAAAGGATCTAGGTGAAGATCCTTTTTGA
TAATCTCATGACCAAAATCCCTTAACGTGAGTTTTCGTTCCACTGAGCGTCAGACCCCGT
AGAAAAGATCAAAGGATCTTCTTGAGATCCTTTTTTTCTGCGCGTAATCTGCTGCTTGCA
AACAAAAAAACCACCGCTACCAGCGGTGGTTTGTTTGCCGGATCAAGAGCTACCAACTC
TTTTTCCGAAGGTAACTGGCTTCAGCAGAGCGCAGATACCAAATACTGTTCTTCTAGTGT
AGCCGTAGTTAGGCCACCACTTCAAGAACTCTGTAGCACCGCCTACATACCTCGCTCTGC
TAATCCTGTTACCAGTGGCTGCTGCCAGTGGCGATAAGTCGTGTCTTACCGGGTTGGACT
CAAGACGATAGTTACCGGATAAGGCGCAGCGGTCGGGCTGAACGGGGGGTTCGTGCACA
CAGCCCAGCTTGGAGCGAACGACCTACACCGAACTGAGATACCTACAGCGTGAGCTATG
AGAAAGCGCCACGCTTCCCGAAGGGAGAAAGGCGGACAGGTATCCGGTAAGCGGCAGG
GTCGGAACAGGAGAGCGCACGAGGGAGCTTCCAGGGGGAAACGCCTGGTATCTTTATAG
TCCTGTCGGGTTTCGCCACCTCTGACTTGAGCGTCGATTTTTGTGATGCTCGTCAGGGGGG
CGGAGCCTATGGAAAAACGCCAGCAACGCGGCCTTTTTACGGTTCCTGGCCTTTTGCTGG
CCTTTTGCTCACATGTTCTTTCCTGCGTTATCCCCTGATTCTGTGGATAACCGTATTACCG
CCTTTGAGTGAGCTGATACCGCTCGCCGCAGCCGAACGACCGAGCGCAGCGAGTCAGTG
AGCGAGGAAGCGGAAGAGCGCCCAATACGCAAACCGCCTCTCCCCGCGCGTTGGCCGAT
TCATTAATGCAGCTGGCACGACAGGTTTCCCGACTGGAAAGCGGGCAGTGAGCGCAACG

CAATTAATGTGAGTTAGCTCACTCATTAGGCACCCCAGGCTTTACACTTTATGCTTCCGGCTCGTATGTTGTGTGGAATTGTGAGCGGATAACAATTTCACACAGGAAACAGCTATGACCATGATTACGCCAAGCGCGAATTCTCCAGGCTTAGAATTCGCTCACTGGCCGTCGTTTTACACCATGATTACGCCAAGCGCGAATTCTCCAGGCTTAGAATTCGCTCACTGGCCGTCGTTTTACAACGTCGTGACTGGGAAAACCCTGGCGTTACCCAACTTAATCGCCTTGCAGCACATCCCCCTTTCGCCAGCTGGCGTAATAGCGAAGAGGCCCGCACCGATCGCCCTTCCCAACAGTTGCGCAGCCTGAATGGCGAATGGGACGCGCCCTGTAGCGGCGCATTAAGCGCGGCGGGTGTGGTGGTTACGCGCAGCGTGACCGCTACACTTGCCAGCGCCCTAGCGCCCGCTCCTTTCGCTTTCTTCCCTTCCTTTCTCGCCACGTTCGCCGGCTTTCCCCGTCAAGCTCTAAATCGGGGGCTCCCTTTAGGGTTCCGATTTAGTGCTTTACGGCACCTCGACCCCAAAAAACTTGATTAGGGTGATGGTTCACGTAGTGGGCCATCGCCCTGATAGACGGTTTTTCGCCCTTTGACGTTGGAGTCCACGTTCTTTAATAGTGGACTCTTGTTCCAAACTGGAACAACACTCAACCCTATCTCGGTCTATTCTTTTGATTTATAAGGGATTTTGCCGATTTCGGCCTATTGGTTAAAAAATGAGCTGATTTAACAAAAATTTAACGCGAATTTTAACAAAATATTAACGCTTACAATTTAG

**Note 2: Scaffold 1033:**

AGACTTCCGGCTTAAGCTCTGAAAGGGTTCTATATCTCCAGGTAGATCTGCTCCCAATGTAACATGCTCGGGACCTACAAGGTGTCAGGATCGAAGATTGCACGACGATGACTTACGGACAGCCGGACGTACTCCCTGAACAATGCGATTCGATATACACGGTGGTGTTCTGTTTGGGCCTTCTGACTCAAAGCCAAGCCTGGATAACAGTGTCCTTCGGGTCCTCCGTGTTATGGAGCACGGCTGCTTAGAGCATTGCCACGGAGCCTTTGAAGGCAGCGAGGGCGTGGCCTCCCGACCGCACTAGCACAACACAGGAGAGGACCGGCGACATACCTGGGTGGAAGTTTCATCGGAATACTCGTCAAACGACTAACCCATCAGCCATCGATCGTATGAATATGTATAATCCATCCGTACCTAGAGTCGCGGGCCAGCATCAACGCATGTGGTAAATTGGTTGGATCCGCGAGCAGTAAGAACCCTTAAATCAAGCTTCCCGGCACAGCGTCAGTGGCGGATACGCGCGATGTCTGCCGTGCTGAAGTAGGGTAAGGGCCCTCCATGCGGACTTCTTGGTAGCTCCAAGAGTGGATTCCCTCGTAAGTTCGGCTACGTCATTTACTGCAGCCTCTGCAAGCGGAGGAACTCCACTATGCACTTAGGGACTACTGATATCAAATTCGCCGCCGAAACCCGCAAGATGGTGCTCTTTAACTGGCCAATACATAGCCCAGTGAAACTGTTCGCATATGGCGAACCTTTCTGCGCCACTTGTTTCGCGCACGTTTAGGAGCTTATTAGCTAAATGAACCAGTTCTCTGGAGTGATACAGACTTGCGGCGTCCCGTAAACCGAGAGTAACTTAACCATAAAGCTGGTGGGTATAGAACATATAGCGATCAGTACGGCCATTTGACGGCAAATACCGTCTGGCTGGCAAGTCGGGCTGATGAAATGCCTGACAGAACTTGAACTGACCAGAAGTGAGCAAAGGCGGTCTCAATTAACACTTTCCTATACGACACGTTGA
